# Application of Psychodynamic Psychotherapy with Cognitive-Behavioral Elements in Substance and Behavioural Addictions

**DOI:** 10.1192/j.eurpsy.2026.10848

**Published:** 2026-07-31

**Authors:** S. Mihaljevic, M. Venus

**Affiliations:** Institute for Public Health Virovitica-Podravina County, Virovitica, Croatia

## Abstract

**Introduction:**

Psychotherapy plays a central role in the comprehensive treatment of addiction, including both substance use and behavioural addictions. When combined with pharmacological interventions or applied on its own, psychotherapy can significantly enhance recovery outcomes and promote long-term behavioural change. This report presents two clinical cases to illustrate the effectiveness of a supportive, psychodynamically-oriented psychotherapeutic approach in the treatment of different forms of addiction.

**Objectives:**

To demonstrate the applicability and effectiveness of supportive, psychodynamically-informed psychotherapy—with integrated cognitive-behavioural elements—in treating both substance-related and behavioural addictions, with or without adjunct pharmacotherapy.

**Methods:**

Two patients were treated over an 24-month period in outpatient individual psychotherapy. Both attended biweekly sessions based on a supportive psychodynamic framework with incorporated cognitive-behavioural strategies.

- **Case 1:** A male patient with a long-standing methamphetamine dependence, comorbid borderline personality traits, and depressive symptoms. Treatment included psychopharmacotherapy (vortioxetine and low-dose olanzapine) and regular urine drug screening.
- **Case 2:** A male patient with gambling disorder, specifically related to frequent betting in land-based bookmakers. He did not receive psychopharmacological treatment and was managed exclusively with psychotherapy.

**Results:**

In both cases, the patients demonstrated significant therapeutic progress. The methamphetamine-dependent patient achieved sustained abstinence, increased insight, and adopted healthier life patterns. Similarly, the patient with gambling disorder maintained full abstinence from gambling for the past 18 months and reported improved emotional regulation and lifestyle stability. In both cases, the therapeutic alliance proved central to maintaining motivation and supporting long-term behavioural change.

**Conclusions:**

These cases illustrate the efficacy of a supportive, psychodynamically-oriented psychotherapeutic approach in the treatment of both substance use and behavioural addictions. Even in the absence of pharmacotherapy, such an approach can lead to significant and lasting improvement, particularly when treatment is consistent and individually tailored. This underscores the importance of psychotherapy as a cornerstone in addiction treatment across diagnostic categories.

**Disclosure of Interest:**

None Declared

